# Efficacy of hearing aid treatment on sound perception and residual hearing preservation in patients with tinnitus and coexisting hearing loss: study protocol for a randomized controlled trial

**DOI:** 10.1186/s13063-022-07014-0

**Published:** 2022-12-27

**Authors:** Peifan Li, Dongmei Tang, Yongzhen Wu, Yanbo Yin, Shan Sun

**Affiliations:** ENT Institute and Otorhinolaryngology, Department of Affiliated Eye and ENT Hospital, Key Laboratory of Hearing Medicine of NHFPC, State Key Laboratory of Medical Neurobiology, Fudan University, Shanghai, 200031 China

**Keywords:** Subjective tinnitus, Sensorineural hearing loss, Hearing aids, Randomized controlled trial

## Abstract

**Background:**

Chronic subjective tinnitus poses significant challenges in clinical practice, and it is usually associated with hearing impairment, particularly with high-frequency sensorineural hearing loss (SNHL). Patients suffering from tinnitus with SNHL experience one of the most severe sensory disabilities, and this has devastating effects on their quality of life. Nowadays, mild to moderate SNHL can be managed with a properly fitted hearing aid (HA) that provides sound amplification, and several studies suggest that HAs may also benefit those with tinnitus. However, inadequate attention has been paid by medical personnel to the impact of HA use in residual hearing protection for patients with tinnitus and coexisting SNHL, and existing evidence is still at a preliminary stage. This study aims to identify and evaluate the efficacy of the use of HAs in both sound perception and residual hearing preservation among patients with tinnitus and coexisting SNHL.

**Methods and design:**

The present study is a prospective, single-center, outcome assessor and data analyst-blinded, randomized, controlled trial. Eligible participants will be recruited and randomly allocated into the HA intervention group and the waiting list control group at a ratio of 1:1. The primary outcome is to evaluate the severity of tinnitus using the Tinnitus Handicap Inventory as a continuous variable at 6 months from randomization. Secondary outcome measures include changes in hearing status and mental states. The trial will last 6 months, with follow-up visits at 3 months and 6 months.

**Discussion:**

This will be the first randomized, controlled trial to identify and evaluate HAs’ efficacy on residual hearing preservation among tinnitus patients with coexisting high-frequency SNHL in China. We are aiming for novelty and generalizability, and strengths of this study are that it will examine the effectiveness of HA in patients with tinnitus and hearing impairment and will further explore the residual hearing protection provided by HA treatment in the tinnitus group.

**Trial registration:**

ClinicalTrials.gov NCT05343026. Registered on April 25, 2022

**Supplementary Information:**

The online version contains supplementary material available at 10.1186/s13063-022-07014-0.

## Background

Subjective tinnitus is defined as an auditory perception without a corresponding auditory stimulus [1], and it has attracted considerable critical attention as a distressing and debilitating audiological condition in clinical practice. Tinnitus increases steadily with age, and the recently reported prevalence of tinnitus is approximately 10 in 100 adults [2, 3], but this is likely to be an underestimation due to poor awareness of the need for timely medical treatment in some patients. Tinnitus can be divided into acute or chronic tinnitus according to its duration. Of particular concern is that about 45 to 78% of patients with chronic tinnitus report bothersome irritability, anxiety, depression, insomnia, and deterioration of social function [4–7]. Current methods for tinnitus management involve cognitive behavioral therapy (CBT), sound therapy, and educational counseling [8]. For most tinnitus cases, the pathophysiological causes are still in dispute [5], so previous specific tinnitus treatments remain substantially heterogeneous [1]. Accordingly, there is an urgent need to develop an effective management strategy for tinnitus.

Moreover, most patients with chronic subjective tinnitus are comorbid with high-frequency sensorineural hearing loss (SNHL) [9]. The occurrence of tinnitus with SNHL presents one of the most common sensory disabilities, leading to a deleterious impact on patients worldwide. It has been recently argued that the generation and maintenance of most tinnitus cases are strongly linked to acquired hearing impairment, especially when the SNHL is associated with distinct neuronal changes in auditory and extra-auditory brain networks [10]. Presbycusis is a mostly irreversible phenomenon that results from the progressive deterioration of hair cell function in the inner ear [11]. With the increased aging of the population, it is predicted that the incidence rate of this condition will continue to increase. However, no strategies are currently available in clinical practice to reverse the progression of this disorder.

Currently, the use of hearing aids (HAs) is widely considered to be an effective instrument for older patients with hearing disabilities. HAs mostly function through sound amplification, and it is noteworthy that the enhanced performance of receiving acoustic information by using HAs is also seen in patients with significant residual hearing [12]. Surprisingly, several studies have shown that HAs might help patients cope with their tinnitus by increasing the external volume to help cover the tinnitus sound [13–16]. However, these previous studies in general do not comprehensively explore the role of HA in the severity or loudness of tinnitus, and there is still little agreement on the relationships between the HA intervention and residual hearing preservation in terms of hearing improvement.

Considering the lack of compelling evidence regarding the extent to which HAs play a role in sound perception and residual hearing preservation among patients with tinnitus and coexisting SNHL, we designed this single-blind, 6-month, randomized, controlled trial with two parallel groups. The first is the HA treatment group, and the second is the waiting list control (WLC) group that will receive non-HA interventions during the study period. We hypothesize that the HA intervention will improve tinnitus patients’ quality of life in terms of improvements in the speech recognition score (SRS), the Tinnitus Handicap Inventory (THI), the Hospital Anxiety and Depression Scale (HADS), and the Athens Insomnia Scale-8 (AIS-8) without affecting residual hearing.

## Methods/design

### Study design and settings

The study protocol will follow the *2013 Standard Protocol Items: Recommendations for Interventional Trials (SPIRIT) Statement* [17] (Additional file 1). This study is a prospective, single-center, outcome assessor and data analyst-blinded, randomized, controlled trial with superiority test in the framework lasting 6 months with an allocation ratio of 1:1 for the intervention and WLC groups. Patients will be recruited from the tinnitus outpatient clinics of the Eye and ENT Hospital of Fudan University in Shanghai, and the trial will be conducted by qualified and well-trained doctors, investigators, and HA technicians using appropriate experimental facilities.

### Participants

Eligible patients who fulfill all of the following criteria will be recruited:

1. Adults aged older than 18 years old and less than 70 years
2. Chronic (> 6 months) subjective tinnitus, unilateral or bilateral
3. Diagnosed with mild high-frequency SNHL (pure tone audiometry (PTA) average of 25–40 dB at 2–8 kHz)
4. Available for 6 months after starting the study to complete the follow-up questionnaires
5. Readiness to participate in the study and sign the informed consent
6. Be covered by public health insurance and be eligible for reimbursement

Patients with any of the following exclusion conditions will not be enrolled:

1. Objective tinnitus
2. Conductive HL
3. Unstable medical history that limits participation
4. Undergoing any other research clinical studies
5. Having used HAs in the past year
6. Unwilling or unable to use HAs daily
7. Alcohol or drug abuse

### Recruitments and randomization

This study will be conducted in the outpatient ENT department of the Eye and ENT Hospital of Fudan University in Shanghai, China. Our institution is a specialized hospital focused on otorhinolaryngology. In addition, we developed an app—the Fudan Tinnitus Relieving System (FTRS)—that will be used for the management, treatment, data collection, and efficacy analysis of tinnitus patients. The participants will be primarily included in the study after being diagnosed with probable chronic subjective tinnitus with bothersome symptoms after the screening procedure. Screening for hearing and tinnitus testing typically consists of PTA, speech audiometry test, and distortion product otoacoustic emissions (DPOAE) along with several widely accepted tinnitus questionnaires such as the THI for tinnitus severity, the visual analog scale (VAS) for tinnitus loudness, and the HADS and AIS-8 for mental health status. The entire test will generally take a mean time of approximately 30 min. Participants will scan the two-dimensional code we supply via their smartphones and will fill out the questionnaires online through the FTRS.

Interested patients will be directed to the research room to receive the necessary details of the study. Before participation, informed and written consent will be obtained from all participants by the principal investigator. The randomization will be stratified by the THI score (≤ 36 or > 36 [18]) based on the baseline assessment in order to avoid an uneven distribution of patients. An independent researcher who is not involved in the recruitment process or outcome measurement will generate a random sequence (http://www.random.org) to randomly assign the participants in a 1:1 allocation to treatment using HAs or to the WLC group. The randomization sequence will be concealed in sequentially numbered sealed and opaque envelopes. Participants will then be notified via telephone or email which group they are allocated to by the staff member who kept the randomization sequence file and who will not participate in any subsequent assessment of the patients. Data analysts, study coordinators, and outcome assessors will remain unaware of the group assignments until the primary analyses are completed.

### Intervention

Following randomization, the participants will be immediately invited to provide personal information including primary demographic data and clinical characteristics. Demographic variables will include gender, age, occupation, residential address, education level, date of onset, and comorbidities. Once participants are randomized and the baseline measurements are complete, the intervention will begin.

Participants in the treatment group are required to wear the HAs for at least 3 h per day and for at least 24 days per month. The HAs will be fitted unilaterally or bilaterally on the side affected by tinnitus, and the HA fitting prescription will be in accordance with the NAL-NL1 procedure [19]. A comfortable and precise level of auditory perception is of the utmost importance in the debugging process of the HA. We will also evaluate the HA performance in the HA treatment group using the Abbreviated Profile of Hearing Aid Benefit (APHAB) questionnaire. The non-HA treatment group is a WLC in which participants will receive the intervention after the waiting period has passed. All participants will receive regular counseling and lifestyle education from physicians during the study period.

**Fig. 1:**
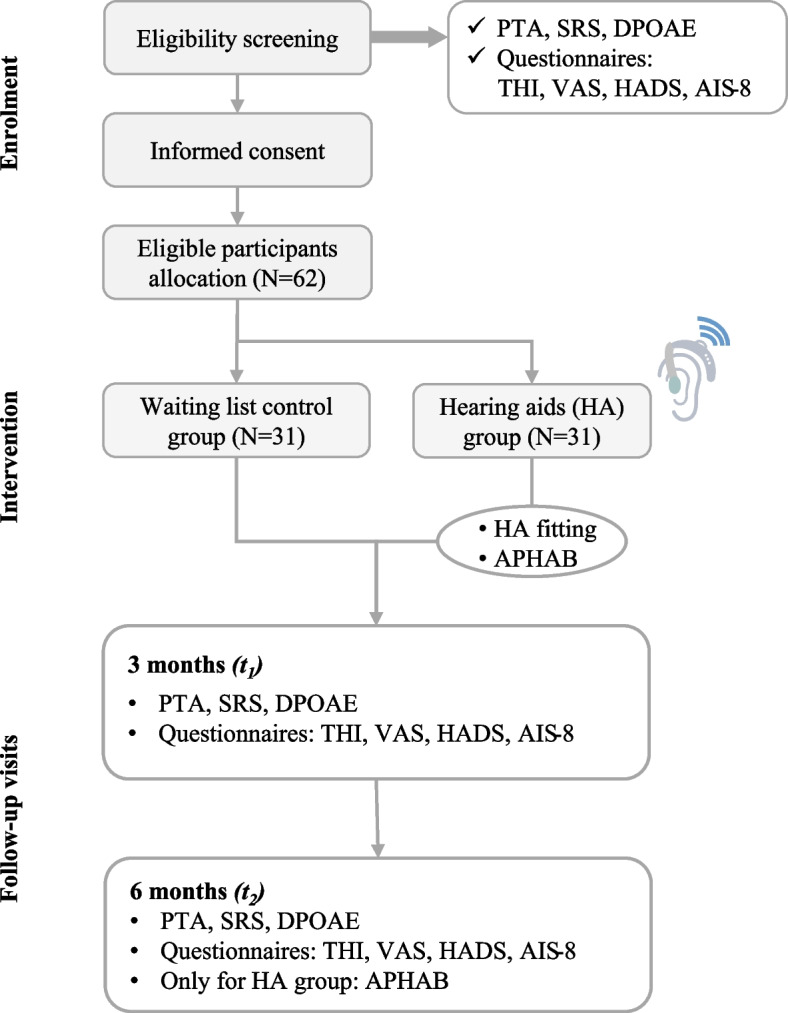
Study flow chart

The flow chart for participant visits and follow-ups is shown in Fig. 1. To assess the outcome efficacy of HAs, all of the participants will undergo follow-up assessments at 3 months and 6 months after the start of the intervention.

### Outcome measurements and follow-ups

#### Primary outcome measure

The primary outcome measure is the change in the THI score [20]. The THI is a self-administered test including 25 questions that determine the degree of function, emotional status, and adverse reactions in the patient with tinnitus. The respondent answers the questions on a 3-point Likert scale with responses of “no” (0 points), “sometimes” (2 points), and “yes” (4 points) yielding an overall score ranging from 0 to 100. The following criteria are used to determine effectiveness [21]: curative = THI score decreased to less than 16 points; significantly effective = THI score decreased by more than 17 points; and ineffective = THI score decreased by less than 17 points, or even increased. The THI has been demonstrated to be a well-known, reliable, and valid clinical tool when evaluating the severity of tinnitus and for assessing the performance of HAs [22]. The proposed study will use the THI Chinese (Mandarin) version [23].

#### Secondary outcome measures

##### SRS

Speech audiometry combines linguistic and acoustic measurements to assess auditory sensitivity and clarity. The SRS test uses a Mandarin monosyllabic word list [24], and participants—who are inside an isolated soundproof chamber—are instructed to repeat each monosyllabic word. The list comprises 25 monosyllabic words, and each word has a score of 4 points on a scale of 100 points such that the scores are given as percentages. A higher score indicates a better level of auditory comprehension and speech recognition.

##### PTA

PTA calculates the average hearing thresholds in decibels (dB) obtained at multiple frequencies. The test is administered in a double-walled soundproof audiometric treatment room with a speaker located 1 m directly in front of the participant. An average hearing threshold above 25 dB at high frequencies (2–8 kHz) is defined as high-frequency SNHL. Participants will be required to undergo PTA at 0.5, 1, 2, and 4 kHz to derive accurate hearing threshold data.

##### DPOAE

The DPOAE test might help determine the mechanism behind tinnitus because it determines whether or not the outer hair cells are overexcited or damaged [25]. DPOAE has the advantage of high-frequency specificity and is an objective and non-invasive measurement for diagnosing hearing impairments [26], which can benefit patients with tinnitus and SNHL.

##### VAS

To measure the subjective perception of tinnitus loudness, we will use the VAS, which requires the patient to mark the severity of their tinnitus on a horizontal scale from 0 to 10. It is easy to administer and widely used as a continuous scale for evaluating tinnitus from asymptomatic to unbearable. The higher the score, the more severe the symptoms.

##### HADS and AIS-8

The HADS and the AIS-8 are two questionnaires for measuring mental status. The HADS consists of 14 items equally divided into two dimensions, namely a depression subscale (HADS-D) and an anxiety subscale (HADS-A) [27, 28]. The grades are valued by scoring as negative (0–7) or positive (8–21). The HADS is easy to use, and the Chinese version’s adequate internal reliability and discriminant validity have been confirmed [29]. Sleep is integral for optimizing brain function and mental states, and the AIS comprises eight items that commonly measure difficulties in falling asleep or maintaining sleep in order to quantify the presence of insomnia [30]. The higher the AIS score, the lower the sleep quality and mental status, and the AIS identifies three types of sleep disorders—no insomnia (score below 4 points), suspicion of insomnia (4–6 points), and insomnia (7–24 points).

##### APHAB

Participants in the HA group will also be asked to complete the APHAB questionnaire in order to additionally evaluate their listening and communication skills. The APHAB consists of 24 questions scored on four six-item subscales [31]. The ease of communication, reverberation, and background noise subscales indicate the characteristics of HA amplification under various environmental conditions, revealing the level of speech comprehension, while the aversiveness to sound subscale indicates the discomfort caused by environmental noise. The subscales of the questionnaire are evaluated separately.

### Time points

**Fig. 2:**
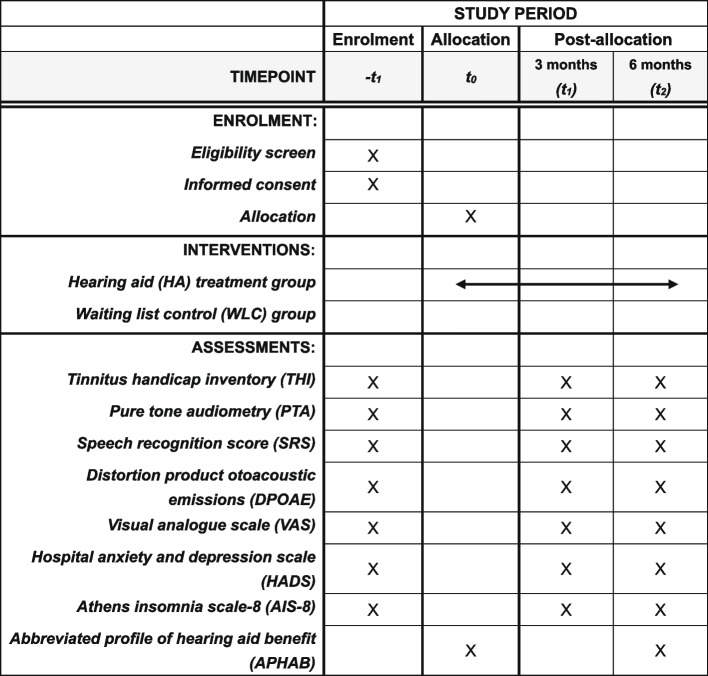
Schedule of enrollment, interventions, and assessments according to the SPIRIT 2013 guidelines

All of the enrolled patients will undergo a clinical evaluation, sign the consent, and record their basic personal information at the baseline measurement. After the stratified randomization, patients will be informed of their group allocation and will correspondingly receive the intervention over the next 6 months. The follow-up visits will be at 3 months after baseline measurement (*t*_1_, mid-way through the intervention) and at 6 months (*t*_2_, the end of the study). The follow-up assessments will comprise the THI, PTA, SRS, DPOAE, VAS, HADS, and AIS-8, and related questionnaires will be developed and distributed in the FTRS. The schedule of enrollment, interventions, and assessments adhering to the SPIRIT 2013 statement is outlined in Fig. 2.

### Adverse events

Adverse events (AEs) are defined as any untoward medical occurrence (such as any abnormal signs and symptoms) in participants in the research regardless of whether or not they are involved in the intervention group. Severe AEs are those leading to hospitalization, discontinuation of treatment, or fatal or life-threatening conditions. AEs will be collected and recorded during the overall trial in our study. There will be two interim analyses carried out to monitor the treatment effects, with further recruitment occurring throughout these interim analyses. One will be carried out when approximately 30 participants have been recruited, and the other will be at 3 months following recruitment. Treatment safety evaluation will be based on AE occurrence and the use of concomitant treatments. Both groups of patients will be allowed to continue their concurrent routinely indicated treatments, and the details of concomitant treatments will be monitored. However, HAs are generally well-tolerated and are not likely to elicit any risk of AEs. An experienced HA-fitting technician will manage the HA intervention if a patient complains about serious side effects.

### Participant withdrawal

The patients will be entitled to withdraw from the study after providing informed consent. We have no stopping guidelines for futility, and if a participant is removed from the study, the precise reasons for withdrawal conditions will be documented and explained. During the overall study time, online contact via a WeChat group and telephone instant messaging will be offered to all of the participants, which will be helpful in managing the trial more effectively and easily.

### Blinding

For this intervention, it is not feasible to blind participants and clinicians regarding the treatment condition owing to the nature of the clinical treatment modalities. However, by separating the intervention and analysis staff, independent investigators in our hospital will perform all follow-up evaluations blinded to the group allocation. We will also attempt to minimize assessment bias and achieve the maximum trial blinding through the following measures: (1) both the data collectors and the assessors will be trained to achieve high interrater reliability, (2) participants and data collectors will be masked as far as possible to block size when collecting the data, and (3) the outcome data assessor will be blinded from randomization until the statistical analyses of the experimental outcomes are completed.

### Trial management and quality control

An independent data monitoring committee will be formed according to the Data Monitoring Committees: Lessons, Ethics, Statistics (DAMOCLES) guidance [32] to oversee the efficacy and safety throughout the trial and will consist of a statistician, an audiologist, an otologist, and an expert in clinical studies.

### Sample size, data collection, and statistical methods

The calculations will be analyzed by the PASS Power Analysis Software (NCSS; Kaysville, UT, USA). According to the power calculations, we assume an *α* of 0.05 and a power of 0.8. The sample size estimation was calculated by the PASS software using the Test for Two Proportions. Based on our clinical experience, feasibility, and the previously reported study [33], with the incidence of a 20% improvement in THI set to 75%, the calculated sample size is 56 participants with a 1:1 allocation. The lost-to-follow-up rate is estimated to be 10%, so we plan to enroll 31 participants in each group.

The data collection will use the FTRS collection platform and the hospital information system to record the answers to the questionnaires and the outcomes of the clinical testing. The questionnaires are designed with inbuilt check-and-skip rules after pretesting by study staff to minimize data-entry errors. The data will be centralized, classified, and protected in long-term storage in the data repository.

Our analysis conforms to the intention-to-treat principle [34]. Descriptive statistics will be used in this study, and IBM SPSS will be used to calculate the means, 95% confidence intervals, ranges, and frequencies. Means ± standard deviations will be used to express the data, and one-way analysis of variance (ANOVA) will be used to compare multiple means. Tinnitus scores from the THI, HADS, and AIS questionnaires will be compared between the HA and WLC groups using Student’s *t*-test with adjustment for multiple comparisons. The chi-square test will be used to test numerical data, and correlations among the study variables will be tested using Pearson’s coefficient. For non-normally distributed variables, the Mann–Whitney *U*-test will be used for between-group comparisons. Adjusted effect size estimates and 95% confidence intervals will be used to determine the statistical significance. A linear model will be used for the analysis with age, gender, and baseline value as covariates. Meanwhile, the effect of treatment will be additionally evaluated by a linear mixed model [35] (if applicable) to account for repeated measures of each outcome. When the proportion of missing values exceeds the predefined threshold of 5%, sensitivity analyses will be conducted using multiple imputation [36] to evaluate the potential impact of the remaining missing values. All of the patient information will be presented anonymously and confidentially throughout the analysis process.

### Ethics and dissemination

This protocol (the original version) was registered with Clinical.Trials.gov (NCT05343026) on 21 April 2022. Ethics approval (Additional file 2) was received from the review board and the ethics committee of the Eye and ENT Hospital of Fudan University (Approval number: 2021173-1). The auditing process involves the review of core trial processes and the verification of source data on a periodic basis, and for additional details, see Additional file 2. The public dissemination of the study results is planned to be achieved in a scientific journal.

## Discussion

SNHL presents an overarching factor and significant confounder in the occurrence and progression of subjective tinnitus. Based on the neuroanatomical studies in tinnitus, it has been shown that the reduced auditory input due to SNHL may lead to neural reorganization and cortex synaptogenesis [37]. Furthermore, most individuals with subjective tinnitus tend to have hearing impairment at high frequencies [38], emphasizing the significance of preserving residual hearing. For auditory comprehension, hair cells in the inner ear are crucial. The hair cells in the apical part of the cochlea detect low-frequency sounds, while those in the basal part detect high-frequency sounds. Because mammalian cochlear hair cells cannot regenerate spontaneously, any damage to the cochlea will have long-term detrimental effects on daily speech and quality of life. Therefore, the protection of residual low-frequency hearing could have significant public health benefits, especially in terms of improved speech understanding [39, 40]. HAs are an efficacious therapy option for patients with hearing impairment, particularly in older patients, and HAs may also benefit patients with tinnitus and SNHL by amplifying the sound and giving partial masking relief.

In addition, a large population of tinnitus patients who suffer from related psychological distress seems to have negative emotional performance [44]. So far, there has been little agreement on how best to manage the mental status of tinnitus patients. The most widely recognized strategy focusing on neuropsychiatric complications is CBT. However, to date, research has not determined whether CBT improves tinnitus’s perceptual characteristics or improves the general quality of life by changing the awareness of tinnitus [41]. Moreover, CBT is ineffective for patients with combined SNHL. Another potential problem with CBT is that it is a time and energy-consuming therapy and requires substantial support from psychiatrists, psychologists, and family connections. HAs, in contrast, are simple, safe, and easy to use, and prior studies have noted the importance of HA use in mental status management in tinnitus patients [14, 42]. To the best of our knowledge, there is no evidence supporting a link between HA treatment and the preservation of residual hearing, and more detailed RCT studies are still required to compensate for the shortcomings of previous studies [16], as well as to further determine the efficacy of HAs in patients suffering from tinnitus with coexisting SNHL.

The present study, which differs from other trials, seeks to comprehensively prove that HAs are effective in patients with tinnitus and hearing impairment and to further explore the residual hearing protection by HA treatment in the intervention group. The strengths of the study rest on several aspects. For instance, we designed the HA treatment as a stand-alone intervention with clearly defined inclusion criteria, including the subjective tinnitus duration and specific PTA thresholds per frequency. Thus, it will be possible to avoid combining the study with other tinnitus treatments and avoid selection bias, which will improve the reliability and credibility of the observation results. Additionally, patients will be recruited from an ENT-specialized hospital, where adequate eligible participants can be obtained. Moreover, the study findings might contribute to profound advances in tinnitus treatments and may serve as a helpful reference for other countries. When the efficacy of the HA can be verified, it may have positive implications in the clinical setting as to its role in alleviating tinnitus and preventing the coexisting SNHL from deteriorating.

This study is not exempt from limitations. For example, most self-report questionnaires are subjective and are prone to reporting bias due to participants’ non-adherence and vague memory. Participants have to be encouraged to answer truthfully, and to some extent, the outcomes provided by caregivers can be added as complementary information. Also, the use of WLC groups has been regarded as an ethical alternative to control groups, but this can potentially pose problems. As a solution to the limitation that participants in the WLC group are stalled at the stage of change regarding readiness, we set the enrollment to be mild and acceptable symptoms, and we will provide periodic health advice to all participants. Another point worth mentioning is that cochlear implant (CI) technology currently effectively restores hearing by stimulating the cochlear nerve [44, 45]. However, although CIs are capable of protecting residual hearing, they are invasive and are prohibitively expensive. Accordingly, early benefits from HA use can be expected in patients with residual hearing. Nevertheless, if the effectiveness is less pronounced with HAs, the use of CIs may be necessary.

To conclude, this will be the first randomized controlled trial to evaluate the efficacy of HAs on sound perception and residual hearing preservation among tinnitus patients with SNHL in China. Despite its limitations, this study is unique and will contribute to knowledge about HAs’ roles in residual hearing preservation in these patients. The results from this trial are expected to provide support for doctors to provide more effective advice in clinical practice.

## Trial status

Participant recruitment for this trial will be initiated in July 2022 and presumably complete in July 2023.

## Supplementary Information

**Additional file 1.** SPIRIT 2013 Checklist.

**Additional file 2.** Ethical approval document.

**Additional file 3.** Funding document.

**Additional file 4.** Informed consent form.

## Data Availability

The study dataset will be available from the corresponding authors upon reasonable request. The results and findings of the trial will be published in peer-reviewed journals and presented at national and international conferences.

## Abbreviations

HA: Hearing aid
WLC: Waiting list control
FTRS: Fudan Tinnitus Relieving System
SPIRIT: Standard Protocol Items: Recommendations for Interventional Trials
THI: Tinnitus Handicap Inventory
SRS: Speech Recognition Score
PTA: Pure-tone audiometry
DPOAE: Distortion product otoacoustic emissions
VAS: Visual analog scale
HADS: Hospital Anxiety and Depression Scale
HADS-D: Hospital Anxiety and Depression Scale – Depression
HADS-A: Hospital Anxiety and Depression Scale – Anxiety
AIS: Athens Insomnia Scale
APHAB: Abbreviated Profile of Hearing Aid Benefit
AE: Adverse event
DAMOCLES: Data Monitoring Committees: Lessons, Ethics, Statistics
CBT: Cognitive behavioral therapy
CI: Cochlear implant
