# Supplementary material for: Efficacy of hearing aid treatment on sound perception and residual hearing preservation in patients with tinnitus and coexisting hearing loss: study protocol for a randomized controlled trial
Source: Trials. 2022 Dec 27;23:1049. doi: 10.1186/s13063-022-07014-0 (PMC9793655; doi:10.1186/s13063-022-07014-0)
Supplement: Supplementary file 2 — Additional file 2. Ethical approval document. [file 13063_2022_7014_MOESM2_ESM.pdf]

复旦大学附属眼耳鼻喉科医院伦理审查批准函

声明：本伦理委员会按照国家卫计委和 NMPA 有关法规组成和工作，其审查和工作过程不受伦理委员会以外任何组织及个人影响

批件号：[2022]伦审字第（2021173-1）号

会议时间	2021-12-23<br>2022-01-20	会议地点	汾阳路 83 号 3 号楼 6 楼第 1 会议室
研究项目名称	助听器对改善双侧听力下降伴慢性主观性耳鸣的疗效评估		
审查文件	初审文件：<br>1. 伦理审查申请表<br>2. 临床研究方案（版本号：Version 1.0；版本日期：2021 年 12 月 03 日）<br>3. 知情同意书（版本号：Version 1.0；版本日期：2021 年 12 月 03 日）<br>4. 病例报告表和其他问诊表（版本号：Version 1.0；版本日期：2021 年 12 月 05 日）<br>5. 主要研究者简历<br>6. 参考文献<br>复审文件：<br>1. 伦理审查申请表<br>2. 临床研究方案（版本号：Version 2.0；版本日期：2022 年 01 月 02 日）<br>3. 知情同意书（版本号：Version 2.0；版本日期：2022 年 01 月 02 日）<br>4. 病例报告表（版本号：Version 2.0；版本日期：2022 年 01 月 02 日）<br>5. 修改内容的清单列表		
研究单位	复旦大学附属眼耳鼻喉科医院		
主要研究者	孙珊		
伦理审查方式	■会议审查 □快速审查		
审查委员	详见“复旦大学附属眼耳鼻喉科医院伦理委员会会议签到表”		
审查意见	1. 2021 年 12 月 23 日会议审查了该项目的研究方案及知情同意书等资料，审查结果为作必要修正后重审，具体意见见伦理审查意见通知函（2021173），2022 年 01 月 20 日会议重审了修改后的研究方案等资料，符合伦理要求，同意进行该项临床研究。<br>2. 伦理委员会对该研究实施过程的年度/定期跟踪审查：■是 □否<br>审查频度为研究批准之日起：□3 个月 □6 个月 ■12 个月<br>伦理委员会有权根据实际进展情况改变年度/定期跟踪审查频度。<br>3. 自批准之日起一年内项目未启动，该批件自动失效。		

主任或副主任委员签字：[illegible]

日期：2022.1.25

复旦大学附属眼耳鼻喉科医院伦理委员会（盖章）

注意：（请仔细阅读）

1. 本伦理委员会批准的项目为涉及人体的生物医学研究，必须严格按照所批最新版本的研究方案和知情同意书开展研究，并遵循国内相关法规指南要求。
2. 凡是涉及人类遗传资源出口或者按照国家规定必须经有关部门专项审批的内容，均需在项目执行前向有关部门申报并获得批准。
3. 本批件可能用于其他中心伦理委员会参考，如果对方案审查存在不同意见，请及时与本伦理委员会沟通。
4. 对已批准的研究方案、知情同意书等材料的任何修改及主要研究者更换等，须及时通知本伦理委员会重新审查，获得批准后执行。
5. 发生严重不良事件及影响研究风险收益比的非预期事件，须及时报告本伦理委员会。
6. 根据伦理委员会对年度/定期跟踪审查频度的意见，无论研究开始与否，请在年度/定期跟踪审查日到期前1个月提出年度/定期跟踪审查的申请。
7. 发现不依从/违反方案情况须及时报告伦理委员会审查。
8. 暂停/提前终止临床研究，请及时通知伦理委员会。
9. 完成研究，须提交结题报告供伦理委员会审查。

地址：上海市汾阳路83号10号楼305室；邮编：200031；电话：021-64377134

The Ethics Committee of the Eye and ENT Hospital of Fudan University
Ethical Approval Form

Declaration: This ethics committee works according to the National Health Commission (NHC) and National Medical Products Administration (NMPA) regulations. The review and work processes of the ethics committee are independent.

EC Ref No. [2022]:2021173-1

<table>
<tr><td>Review Date</td><td>2021-12-23
2022-01-20</td><td>Review Address</td><td>6th Floor Room 1, Building 3, No. 83, Fen Yang Road, Xuhui District, Shanghai, China</td></tr>
<tr><td>Project Title</td><td colspan="3">Efficacy of hearing-aid treatment for patients with tinnitus and co-existing hearing loss</td></tr>
<tr><td>Documents for Review</td><td colspan="3">Initial review:
1. Ethical Approval Application Form
2. Clinical study protocol (Version 1.0; 2021-12-03)
3. Informed consent (Version 1.0; 2021-12-03)
4. Case report form and questionnaires (Version 1.0; 2021-12-05)
5. Resume of the principal investigator
6. References
Second review:
1. Ethical Approval Application Form
2. Clinical study protocol (Version 2.0; 2022-01-02)
3. Informed consent (Version 2.0; 2022-01-02)
4. Case report form (Version 2.0; 2022-01-02)
5. List of revised contents</td></tr>
<tr><td>Affiliation of Research</td><td colspan="3">The Eye and ENT Hospital of Fudan University</td></tr>
<tr><td>Principal Investigator</td><td colspan="3">Shan Sun</td></tr>
<tr><td>Review Mode</td><td colspan="3">■Conference review ☐Express review</td></tr>
<tr><td>Review Members</td><td colspan="3">See the sign-in sheets for details.</td></tr>
<tr><td>Review Comments</td><td colspan="3">1. On 23 December 2021, the committee reviewed the study protocol. The comment was to amend the protocol outlined in the ethics review opinion letter (2021173). On 20 January 2022, the committee reexamined the amended protocol and formally APPROVED this study to proceed under the current protocol.
2. Annual/periodic follow-up reviews by the Ethics Committee: ■Yes ☐No
Review frequency from the approval date: ☐3 months ☐6 months ■12 months
It is up to the Ethics Committee to determine whether annual/periodic follow-up reviews should be changed based on actual study progress.
3. If the project is not initiated within one year from the approval date, this approval will automatically expire.</td></tr>
<tr><td colspan="4" align="right">Signature of the Director: Xiaofen Mo
Date: 2022.1.25
The Ethics Committee of the Eye and ENT Hospital of Fudan University (sealed)</td></tr>
</table>

NOTES: PLEASE READ CAREFULLY

1. The research projects approved by this Ethics Committee involve biomedical research involving human subjects and must be carried out strictly following the latest approved research protocol and informed consent form, as well as in compliance with the relevant domestic regulatory guidelines.
2. Before implementing a project that involves the export of human genetic resources or requires specific approval by relevant authorities following national regulations, the project must be declared to and approved by the relevant authorities.
3. The ethics committees of other institutions may be able to use this approval for reference purposes. If there is a different opinion regarding the protocol review, please contact the ethics committee as soon as possible.
4. Before incorporating any changes into the approved research protocol or informed consent form, the Ethics Committee must be notified on time for re-examination and approval.
5. This Ethics Committee should be notified of any severe adverse events or unintended events affecting the risk-benefit ratio of the study promptly.
6. According to the Ethics Committee's opinion regarding the frequency of annual/periodic follow-up reviews, please apply one month before the annual/periodic follow-up review date is due, regardless of whether the study has begun.
7. If there is any non-compliance with the protocol or breach of law, it must be reported immediately to the Ethics Committee.
8. In the event of a suspension or early termination of a clinical study, please inform the Ethics Committee immediately.
9. Upon completing the study, a final report must be submitted to the Ethics Committee for review.

Address: Room 305, Building 10, No. 83 Fen Yang Road, Shanghai, 200031, China;
Telephone Number: +86-021-64377134
