# Supplementary material for: Efficacy of hearing aid treatment on sound perception and residual hearing preservation in patients with tinnitus and coexisting hearing loss: study protocol for a randomized controlled trial
Source: Trials. 2022 Dec 27;23:1049. doi: 10.1186/s13063-022-07014-0 (PMC9793655; doi:10.1186/s13063-022-07014-0)
Supplement: Supplementary file 3 — Additional file 3. Funding document. [file 13063_2022_7014_MOESM3_ESM.pdf]

上海市卫生健康委员会文件

沪卫科教〔2020〕38号

关于公布
老龄化和妇儿健康研究专项立项项目的通知

有关单位:

为全面贯彻落实《全国医疗卫生服务体系规划(2015-2020)》《“十三五”卫生与健康科技创新专项规划》《上海市医学科技创新发展“十三五”规划》等要求，进一步提高老年人、妇女儿童等重点人群健康，我委开展了老龄化和妇儿健康研究专项。

经专家评审以及我委预算安排，现决定将“基于人机互动的老年认知衰退早期干预训练的随机对照研究”等62项项目(名单见附件)列入老龄化和妇儿健康研究专项，资助经费按项目申请资助数额资助，最高不超过50万元，最终资助经费以预算评审结果为准，各单位对立项课题可根据实际情况予以配套支持。

老龄化和妇儿健康研究专项研究周期为 3 年，请各项目承担单位遵循医学伦理、生物安全以及知识产权的有关要求开展研究，根据计划任务书及相关财务制度的要求，加强专项经费管理，专款专用，规范资金使用，加强对项目的管理和支持力度，确保项目按既定目标实施，并按我委有关规定定期汇报进展情况。

特此通知。

附件：老龄化和妇儿健康研究专项立项项目名单

上海市卫生健康委员会

2020 年 8 月 5 日

（此件公开发布）

上海市老龄化和妇儿健康研究专项
计划任务书

项目编号：2020YJZX0110

项目名称：老年性听觉功能减退三级防控关键技术研究

项目承担单位（盖章）：复旦大学附属眼耳鼻喉科医院

项目负责人：孙珊

项目年限：2020 年 9 月 至 2023 年 8 月

填报日期：2020 年 08 月 18 日

项目责任单位意见

法定代表人签字：周行涛

单位盖章：

二〇　　年　　月　　日

主管部门意见

单位盖章：

二〇　　年　　月　　日

上海市卫生健康委意见

同意

单位盖章：

二〇　　年　　月　　日

Shanghai Municipal Health Commission Document

Shanghai Medical and Health Science and Technology No. [2020]38

Announcement

Grant for the Aging, Women, and Children Health Research Projects

Relevant units:

Our committee has carried out special research projects to fully implement the requirements of the regulations (the National Health Plan 2005-2020) (the 13th Five-Year Plan for the Health Scientific and Technological Innovation) (the 13th Five-Year Plan for Science and Technology Development in Shanghai) and to improve the healthcare of key populations:the elderly, women, and children.

Sixty-two projects have been acknowledged in the Special Program for Research on Aging and Women's and Children's Health after expert review and budgetary arrangements. The amount of grant, up to RMB 500,000, is determined based on how much the projects request. The budget evaluation determines the amount of funding. Units could support the established projects based on the actual situation.

The research duration on aging and women's and children's health will be 3 years. Applicants must adhere to the medical ethics, biosafety, and intellectual property rights requirements. Furthermore, the accountability

units should pay attention to the management of funds based on the mission statement and the financial requirement of program. The units also should use funds specifically and appropriately. Meanwhile, they shoud strengthen the management and support of the projects following the relevant regulations of our commission.

Hereby notify the above.

Attachment: Projects on aging and women's and children's health.

Shanghai Municipal Health Commission (Sealed)

5 August 2020

(Publicly available)

the Shanghai Health Research project
for the Aging, Women, and Children

Project number: 2020YJZX0110

Project name:

The study of critical technologies for the prevention and treatment of age-related hearing loss

Project Accountability Unit (stamped):

Eye, Ear, Nose, and Throat Hospital of Fudan University

Project Leader: Shan Sun

Project duration: September 2020 - August 2023

Declaration date: 18 August 2020

The Opinion of the Accountability Unit of the Project<br><br><br><br><br>Legal representative (signature)<br>Unit seal
The Opinion of the Administration Office<br><br><br><br><br>Shanghai Medical College of Fudan University<br>Unit seal
The Opinion of the Shanghai Municipal Health Commission:<br><br>Approved.<br><br><br><br>Unit seal
