# Supplementary material for: Efficacy of hearing aid treatment on sound perception and residual hearing preservation in patients with tinnitus and coexisting hearing loss: study protocol for a randomized controlled trial
Source: Trials. 2022 Dec 27;23:1049. doi: 10.1186/s13063-022-07014-0 (PMC9793655; doi:10.1186/s13063-022-07014-0)
Supplement: Supplementary file 4 — Additional file 4. Informed consent form. [file 13063_2022_7014_MOESM4_ESM.pdf]

Participant Informed Consent Form

Study Title: Efficacy of hearing-aid treatment on sound perception and residual hearing preservation in patients with tinnitus and coexisting hearing loss: study protocol for a randomized controlled trial

Clinical trial registration: NCT05343026

Dear Sir or Madam,

We are pleased to invite you to participate in our study. It is your decision whether or not to participate. Please refer to the following participant information document for further information on why we are conducting this study, your involvement in the study, and the benefits and risks you may encounter. If you have any further questions regarding this information, do not hesitate to contact us. It is also advised that you discuss the study with your family and friends before deciding on any course of action.

1. Introduction

Background: The experience of tinnitus can be very distressing, as it involves hearing unwanted sounds, such as ringing or buzzing. Sleep, mood, and concentration may be adversely affected as a result of tinnitus. There is a need to ensure that tinnitus clinical services are readily available throughout the country, however this is difficult to achieve given the burdens on the current health care system. In order to effectively manage the health care burden associated with tinnitus, we must develop new ways of helping those suffering from the condition. It is now possible to manage mild to moderate hearing loss with a properly fitted hearing aid (HA) that provides sound amplification, and several studies suggest that HAs may also provide benefits to individuals with tinnitus. For patients with hearing impairment, particularly those who are aging, hearing aids are an effective therapy option. By amplifying the sound and providing partial masking relief, the HA may also benefit patients with tinnitus and hearing loss.

Aims：There has been insufficient attention paid by medical personnel to the impact of HA use in residual hearing protection for patients who have tinnitus and coexisting

hearing loss, and the evidence is only at a preliminary stage. This study is intended to provide findings that may contribute to profound advances in the treatment of tinnitus and may serve as a useful reference for other countries. In the clinical setting, HA may have positive implications in terms of alleviating tinnitus and preventing the coexisting hearing loss from worsening, if the efficacy of the device can be verified. In the event that you decide to participate, we would appreciate if you could complete a questionnaire. In order to ensure the confidentiality of all information recorded, it will be encrypted and stored in a secure manner.

2. Methods

There will be no tissue or blood samples involved in this study. This trial was registered with Clinical.Trials.gov on 21 April 2022. Ethics approval was received from the review board and the ethics committee of the Eye and ENT Hospital of Fudan University. The specific steps are described below:

2.1 the screening procedure: participants will be primarily included in the study after being diagnosed with probable chronic subjective tinnitus with bothersome symptoms. Screening for hearing and tinnitus testing typically consists of pure tone audiometry (PTA), speech audiometry test, and distortion product otoacoustic emissions along with several widely accepted tinnitus questionnaires.

2.2 Following randomization, the participants will be assigned in a 1:1 allocation to treatment using HAs or to the waiting list control group. Participants in the HA group are required to wear the HAs for at least 3 hours per day and for at least 24 days per month. The non-HA treatment group is a WLC in which participants will receive the intervention after the waiting period has passed. All participants will receive regular counselling and lifestyle education from physicians during the study period.

2.3 The follow-up visits will be at 3 months after baseline measurement (t1, mid-way through the intervention) and at 6 months (t2, the end of the study).

2.4 As part of the project, data will be recorded using written documentation and software. Participants will be identified by a number which appears on the records. A separate record of participants' names and their allocated number will be kept

secure during the project and will be destroyed immediately after the project has been completed, along with all other personal information which may identify individuals.

3. Possible benefits and risks

This study is non-invasive and will not affect your concurrent treatment, nor will it adversely affect your physical, psychological or social relationships. For all participants, routine counselling and mental health care are offered free of charge and without the need for registration at an outpatient visit. All study procedures are under the supervision of the Eye, Ear, Nose and Throat Hospital of Fudan University. You can contact the study doctor with any questions you may have during the study process.

As part of this study, you are voluntarily participating, and you may decline to participate or withdraw from the research at any time without experiencing any disadvantages. You are welcome to contact Dr. Tang with any questions or concerns you may have during or following your participation in this research.

Please sign your name below to provide your consent to participate in this study:

Declaration by participant: I have read and understood the participant information form. It has been sufficient time for me to consider whether or not to participate in this study. I am satisfied with the answers I have received regarding the study and I have a copy of the consent and information sheet.

I hereby consent to take part in this study.

Participant's Name: ______________; Date: _______________

Declaration by the researcher: I have given an explanation of the research project to the participant and have given informed consent to participate.

Researcher's Name: ______________; Date: _______________
